# Loss of DARPP-32 and calbindin in multiple system atrophy

**DOI:** 10.1007/s00702-013-1039-4

**Published:** 2013-05-29

**Authors:** Hideki Hayakawa, Makiko Nagai, Aya Kawanami, Yasuto Nakata, Tomoko Nihira, Mieko Ogino, Masahiko Takada, Takaomi Saido, Jiro Takano, Makoto Saegusa, Tetsuo Mikami, Junichi Hamada, Kazutoshi Nishiyama, Hideki Mochizuki, Yoshikuni Mizuno

**Affiliations:** 1 Department of Neuroregenerative Medicine, Kitasato University School of Medicine, 1-15-1 Kitasato, Minami-ku, Sagamihara, Kanagawa 252-0374 Japan; 2 Department of Neurology, Kitasato University School of Medicine, Sagamihara, Kanagawa Japan; 3 Primate Research Institute, Kyoto University, Inuayama, Aichi Japan; 4 Laboratory for Proteolytic Science, RIKEN Brain Science Institute, Wako, Saitama Japan; 5 Department of Pathology, Kitasato University School of Medicine, Sagamihara, Kanagawa Japan; 6 Department of Neurology, Osaka University School of Medicine, Suita, Osaka Japan

**Keywords:** Multiple system atrophy, DARPP-32, Calbindin-D 28k, Glial cytoplasmic inclusion, Pathogenesis

## Abstract

We evaluated the immunohistochemical intensities of α-synuclein, phosphorylated α-synuclein (p-syn), dopamine- and cAMP-regulated phosphoprotein of 32 kDa (DARPP-32), calbindin-D 28k, calpain-cleaved carboxy-terminal 150-kDa spectrin fragment, and tyrosine hydroxylase in multiple system atrophy (MSA). The caudate head, anterior putamen, posterior putamen, substantia nigra, pontine nucleus, and cerebellar cortex from six MSA brains, six age-matched disease control brains (amyotrophic lateral sclerosis), and five control brains were processed for immunostaining by standard methods. Immunostaining for α-synuclein, p-syn, or both was increased in all areas examined in oligodendrocytes in MSA. Immunostaining for DARPP-32 and calbindin-D 28k was most prominently decreased in the posterior putamen, where neuronal loss was most prominent. Immunostaining for DARPP-32 and calbindin-D 28k was also diminished in the anterior putamen and caudate head, where neuronal loss was less prominent or absent. Calbindin immunostaining was also decreased in the dorsal tier of the substantia nigra and cerebellar cortex. Loss of immunostaining for DARPP-32 and calbindin-D 28k compared with that of neurons indicates calcium toxicity and disturbance of the phosphorylated state of proteins as relatively early events in the pathogenesis of MSA.

## Introduction

Multiple system atrophy (MSA), first described by Adams et al. (1964), is a progressive neurological disorder for which no symptomatic or neuroprotective treatment is presently available. The etiology and pathogenesis of this disorder are unknown. The striatonigral, pontocerebellar, and autonomic nervous systems are almost always involved; however, other systems such as the thalamus, frontal cortex, and pyramidal systems may also be involved (Wenning et al. 1997; Ozawa et al. 2004). Glial inclusions consisting of α-synuclein have been acknowledged as a pathognomonic hallmark of MSA (Papp et al. 1989; Nakazato et al. 1990). However, the molecular mechanism of α-synuclein accumulation is unknown.

Calbindin-D 28k is a calcium-binding protein expressed in many organs, including the brain (Séquier et al. 1988). One of the important functions of calbindin is buffering of calcium entry on stimulation of glutamate receptors. The intracytoplasmic concentration of calcium is approximately one-hundredth that of extracellular calcium. Therefore, derangement of calbindin can initiate a degenerative process caused by calcium toxicity.

Calbindin-D 28k was reported to be unevenly distributed in the striatum, i.e., higher in the caudate head and anterior putamen and lower in the posterior putamen (Karachi et al. 2002). The striatal lesions in MSA are less prominent in the caudate head and anterior putamen and more prominent in the posterior putamen (Ozawa et al. 2004). Therefore, calbindin-D 28k might be a factor in the pathogenesis of MSA. If the function of calbindin is decreased or calcium entry is increased, calpain, a calcium-activated proteinase, might be activated. Spectrin is one of the substrates for calpain, and the breakdown product, a 150-kDa spectrin fragment, is generated when calpain is activated (Taniguchi et al. 2001). To observe calpain activation, we performed immunostaining for the 150-kDa spectrin fragment. Damier et al. (1999) reported marked reduction in immunostaining for calbindin-D 28k in the ventrolateral tier of the substantia nigra, which is the most severely involved part in Parkinson’s disease (PD), and suggested that this might contribute to the neurodegeneration in PD.

## Materials and methods

We used immunohistochemical analysis to study six autopsy cases of MSA, six age-matched disease control cases of amyotrophic lateral sclerosis (ALS), and five control cases. The study was approved by the ethics committee of the Kitasato University School of Medicine.

Brain tissue samples were fixed postmortem in 10 % formalin and embedded in paraffin. We focused on the following six areas in the brain: the posterior putamen, anterior putamen, caudate head, substantia nigra, pontine nucleus, and cerebellar cortex. The posterior putamen samples were obtained from the plane behind the anterior commissure, where the globus pallidus externa and the interna were clearly observed together with the putamen. The anterior putamen and caudate head samples were obtained from the plane anterior to the anterior commissure, where the caudate head and anterior putamen with the internal capsule in between, were observed.

The brain sections (6-μm thickness) were deparaffinized, rehydrated, and autoclaved in 10-mM citric acid buffer (pH 6.0) for 20 min at 121 °C for antigen retrieval. Following pretreatment with 3 % H_2_O_2_/methanol for 10 min to eliminate endogenous peroxidase activity, the sections were incubated for 24 h with primary antibodies diluted in phosphate-buffered saline (PBS) containing 10 % blocking at 4 °C (Block Ace; Yukijirushi-Nyugyo Co., Sapporo, Japan). The primary antibodies used were as follows: mouse anti-α-synuclein (clone LB509, diluted at 1:500; Zymed Laboratories, South San Francisco, CA, USA), anti-p-syn (Ser-129, 1:10,000; Wako, Osaka, Japan), rabbit anti-dopamine- and cAMP-regulated phosphoprotein of 32 kDa (DARPP-32) (1:2,000, Millipore; MA, USA), rabbit anti-calbindin-D 28k (1:500, Millipore), rabbit anti-calpain-cleaved carboxy-terminal 150-kDa spectrin fragment (1:200) (Saido et al. 1993), and rabbit anti-tyrosine hydroxylase (TH) (1:1,000; Calbiochem, CA, USA).

After treatment with a primary antibody, the sections were incubated with the biotinylated second antibody (anti-mouse, anti-rabbit IgG antibody, 1:500; Vector Laboratories Inc, CA, USA) at room temperature for 1 h. They were then incubated in an avidin–biotin peroxidase complex (Vector Laboratories Inc.) for 1 h. Visualization of the immunostaining was performed by incubation with a working solution of 3,3-diamino benzidine (Sigma-Aldrich, MO, USA) for 5 min. The sections were washed with PBS, subjected to nuclear staining with hematoxylin, dehydrated in graded ethanol solutions, cleared in xylene, and protected by a coverslip.

**Fig. 1:**
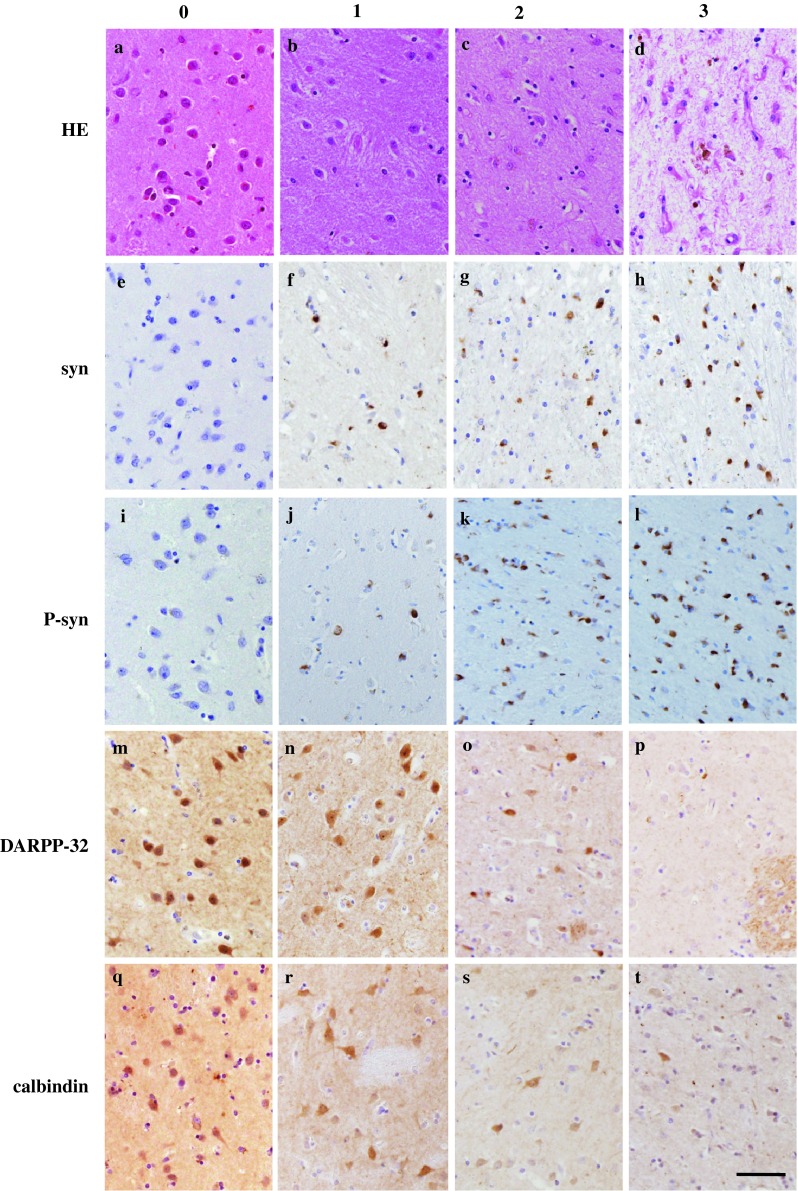
Examples of semiquantitative evaluation of the histological analysis and immunostaining. “0” represents a normal state. Examples from the control cases. “1” represents mild loss, “2” moderate loss, and “3” marked loss. Examples from patients with MSA. All the images are from the striatum, HE staining **a**–**d**, synuclein staining **e**–**h**, P-syn staining **i**–**l**, DARPP-32 staining **m**–**p**, and calbindin staining **q**–**t**. The *calibration bar* indicates 50 μm

The degree of neuronal degeneration in hematoxylin and eosin (HE) staining was evaluated semiqualitatively by scoring from 0 to 3: 0, normal; 1, mild; 2, moderate; and 3, marked loss of neurons. Immunostaining for α-synuclein and p-syn was also evaluated semiquantitatively from 0 to 3: 0, no immunostaining; 1, mild; 2, moderate; and 3, marked increase in immunostaining. The degree of immunostaining for DARPP-32, calbindin-D 28k, the 150-kDa spectrin fragment, and TH was also evaluated semiquantitatively from 0 to 3: 0, normal immunostaining; 1, mild; 2, moderate; and 3, marked decrease in immunostaining. Semiquantitative evaluation of the staining was performed by three investigators (H.H., M.N., and Y.M.). Sample scores that differed were averaged and the results were rounded to the nearest integral number. Representative examples of immunostaining so scaled are shown in Fig. 1.

## Results

### Clinical summary

Clinical summaries of the cases studied are listed in Table 1. All the disease control cases had ALS. MSA 1 (female) developed cerebellar ataxia at age 45 years, which was followed by Parkinsonism, pyramidal signs, autonomic failure, and sleep apnea, and she died at 58 years due to bronchopneumonia. MSA 2 (male) developed Parkinsonism at 69 years of age, which was followed by autonomic failure and pyramidal signs, and he died at 74 years of age due to sputum obstruction of the bronchus. MSA 3 (male) developed dysuria and Parkinsonism at 54 years of age, which was followed by cerebellar ataxia, autonomic failure, sleep apnea, and pyramidal signs. He developed anoxic encephalopathy 10 years after the onset from sleep apnea, was in a vegetable state thereafter, and died at 67 years of age due to respiratory failure. MSA 4 (male) developed Parkinsonism at 64 years of age, which was followed by autonomic failure and pyramidal signs, and the patient died at 67 years of age due to a left frontal hemorrhage. MSA 5 (male) developed cerebellar ataxia at 48 years of age, which was followed by autonomic failure and pyramidal signs, and he died at 58 years of age due to bronchopneumonia. MSA 6 (male) developed cerebellar ataxia at 69 years of age, which was followed by autonomic failure and pyramidal signs, and he died due to an unknown cause at 73 years of age.

**Table 1:** Clinical synopsis of the control, ALS, and MSA cases studied

Autopsy number	Sex	Age at onset (years)	Age at death (years)	Duration of illness (years)	Initial symptom	Neurological findings	Cause of death	Time to autopsy (h)
Control 1	M		77				Adenocarcinoma	3
Control 2	F		62				Plasma cell myeloma	3
Control 3	M		85				Pulmonary thrombosis	2.5
Control 4	M		78				Neuroendocrine carcinoma	3.5
Control 5	M		60				Small cell carcinoma	3
ALS 1	F	69	72	3	Gait disturbance	Paraplegia, bulbar palsy	Respiratory failure	12
ALS 2	M	54	56	2	Left leg weakness	Paraplegia, bulbar palsy	Respiratory failure	13.5
ALS 3	F	77	80	2	Dysarthria	Paraplegia, bulbar palsy, dementia	Respiratory failure	2.3
ALS 4	M	57	58	1	Right hand weakness	Paraplegia, bulbar palsy	Respiratory failure	5
ALS 5	F	70	73	3	Right arm weakness	Paraplegia, bulbar palsy, dementia	Respiratory failure	14
ALS 6	F	63	65	2.5	Gait disturbance	Paraplegia, bulbar palsy	Respiratory failure	6
MSA 1	F	45	58	13	Gait disturbance	Parkinsonism, ataxia, autonomic,	Broncho-pneumonia	9.5
						Pyramidal		
MSA 2	M	69	74	5	Akinesia	Parkinsonism, autonomic,	Sputum obstruction	9
						Pyramidal, frontal	Of the bronchus	
MSA 3	M	54	67	13	Dysuria	Parkinsonism, ataxia	Respiratory failure	16
						Autonomic, pyramidal		
MSA 4	M	64	67	3	Gait disturbance	Parkinsonism, autonomic, pyramidal	Left frontal hemorrhage	15
MSA 5	M	48	58	10	Gait disturbance	Ataxia, autonomic, pyramidal	Broncho-pneumonia	2
MSA 6	M	69	73	4	Ataxia	Ataxia, autonomic, pyramidal	Unknown	5

### Immunohistochemical studies

#### Control and ALS cases

No neuronal loss was noted in any of the striatal regions, substantia nigra, pons, and cerebellar cortices. None of the control and ALS cases had α-synuclein or p-syn immunostaining. Immunostaining for DARPP-32 was positive in the striatal neurons, but not in the nigral, pontine, and cerebellar cortical neurons. No loss of striatal immunostaining against DARPP-32 was noted in the control and ALS cases. Some caudate–putamen gradients in the immunostaining of DARPP-32 were noted in two of the five control cases; immunohistochemistry staining of the caudate neurons was evident in the control and ALS cases, whereas that in the anterior and posterior putamen was somewhat less evident in two of the five control cases. Immunostaining results for calbindin-D 28k was positive in the striatal neurons, but not in the nigral neurons, except for those in the dorsal tier along the lower border of the red nucleus. No loss of immunostaining for calbindin-D 28k was noted in the striatum and substantia nigra in any of the control and ALS cases. No clear gradient in the immunostaining of calbindin-D 28k was noted in the striatum. Immunostaining against calbindin-D 28k was absent in the pontine neurons, but Purkinje cells in the cerebellar cortices were very well stained. Immunostaining results against the 150-kDa spectrin fragment were negative in neuronal cell bodies in the striatum, substantia nigra, pons, and cerebellar cortices in all the control and ALS cases. Immunostaining against TH was normal in the striatum and substantia nigra in all the control and ALS cases.

#### MSA cases

**Fig. 2:**
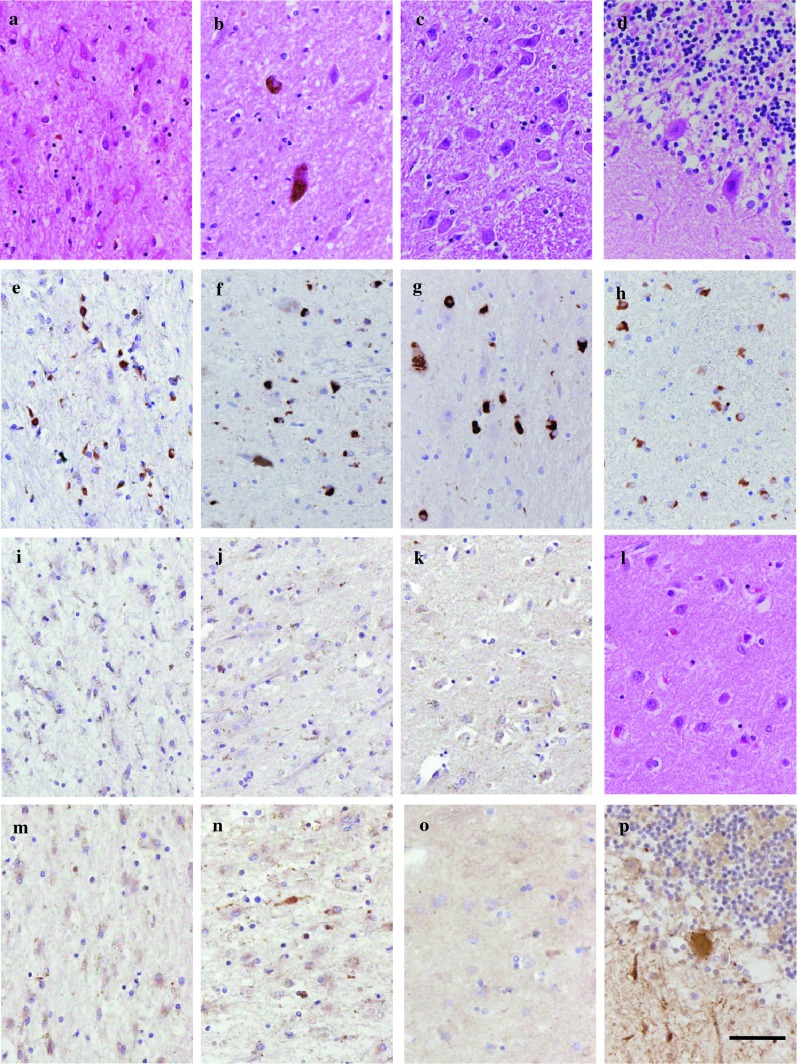
Loss of immunostaining in MSA. All the images are from case MSA 2. HE staining shows marked neuronal loss in the posterior putamen **a**, moderate neuronal loss in the substantia nigra **b**, and moderate loss in the cerebellar cortex **d**. Only mild neuronal loss is noted in the pontine nucleus **c**. Immunostaining with p-syn reveals a marked increase in glial inclusions in the posterior putamen **e**, substantia nigra **f**, pontine nucleus **g**, and cerebellar cortex **h**. Marked decline in intensities with DARPP-32 immunostaining are noted in the posterior putamen **i**, anterior putamen **j**, and caudate head **k**. Neuronal loss in the caudate nucleus is not noticeable **l**, despite marked loss of intensities in DARPP-32 (k) and calbindin-D 28k immunostaining **o**. No neurons show immunostaining with calbindin-D 28k in the posterior putamen **m**. Some neurons in the anterior putamen, caudate head **o**, and cerebellar cortex show immunostaining with calbindin-D 28k **p**. The *calibration bar* indicates 50 μm

The posterior putamen showed extensive loss of neurons in MSA cases 1–4 (Table 2) by HE staining. The anterior putamen showed moderate and mild neurodegeneration in two and one, respectively, of the five cases; the caudate head showed only mild neurodegeneration in three of the five cases. MSA 3 was complicated by anoxic encephalopathy. Anoxic changes were observed in the cortical neurons adjacent to the putamen and in the globus pallidus; however, we could evaluate the immunohistochemical changes in this case because the changes were thought to be caused by MSA and anoxic encephalopathy. Brain stem structures in this case were easily identified. In this case, no specimens of the anterior putamen and caudate head remained. Striatal lesions were minimal in MSA 5 and absent in MSA 6. In these cases, the substantia nigra and pontocerebellar system were markedly (MSA 5) or moderately involved (MSA 6) (Table 3), and p-syn was increased in oligodendrocytes in all areas examined. Thus, they fulfilled the pathological criteria for MSA. In the other four cases of MSA, excluding MSA 4, increased accumulation of p-syn in oligodendrocytes was observed in both the nigrostriatal and pontocerebellar systems (Fig. 2). In MSA 4, immunostaining for p-syn was negative in the putamen but positive in the cerebellum, whereas that for α-synuclein was positive in both regions (Fig. 2; Table 2). None of the cases had Lewy bodies or tau inclusions. P-syn accumulation was observed mainly in oligodendrocytes; however, some astrocytes also accumulated α-synuclein. Nigral neurons were either markedly or moderately reduced in the pars compacta. The number of neurons in the pontine nucleus was decreased, and the remaining neurons showed atrophic changes. Alpha-synuclein and p-syn were observed in the white matter in the pyramidal tracts and transverse pontine fibers. The cerebellar cortex showed either marked or moderate neuronal loss in the Purkinje cells. The dentate nucleus was retained. Alpha-synuclein and p-syn were accumulated in the oligodendrocytes in the granular cell layers and the subcortical white matter in the cerebellum.

**Table 2:** Immunostaining Intensity in the caudate head and putamen

Case no. and Dx	Posterior putamen						Anterior putamen						Caudate head					
	HE	Syn	P-syn	D-32	CALB	TH	HE	Syn	P-syn	D-32	CALB	TH	HE	Syn	P-syn	D-32	CALB	TH
Control 1	0	0	0	2	0	0	0	0	0	2	0	0	0	0	0	0	0	0
Control 2	0	0	0	1	0	0	0	0	0	0	0	0	0	0	0	0	0	0
Control 3	0	0	0	0	0	0	0	0	0	0	0	0	0	0	0	0	0	0
Control 4	0	0	0	2	0	0	0	0	0	2	1	0	0	0	0	0	0	0
Control 5	0	0	0	0	0	0	0	0	0	0	0	0	0	0	0	0	0	0
ALS 1	0	0	0	0	1	0	0	0	0	0	1	0	0	0	0	0	1	0
ALS 2	0	0	0	0	0	0	0	0	0	0	0	0	0	0	0	0	0	0
ALS 3	0	0	0	0	0	0	0	0	0	0	0	0	0	0	0	0	0	0
ALS 4	0	0	0	1	0	0	0	0	0	0	0	0	0	0	0	0	0	0
ALS 5	0	0	0	0	0	0	0	0	0	0	0	0	0	0	0	0	0	0
ALS 6	0	0	0	0	0	0	0	0	0	0	0	0	0	0	0	0	0	0
MSA 1	3	3	3	3	2	3	1	1	3	3	2	3	1	1	3	3	2	3
MSA 2	3	3	3	3	3	3	2	3	3	3	2	2	1	0	3	3	2	2
MSA 3	3	1	2	3	3	3	NS	NS	NS	NS	NS	NS	NS	NS	NS	NS	NS	NS
MSA 4	3	3	0	3	3	3	2	3	0	3	3	3	1	1	0	3	3	2
MSA 5	1	3	3	3	3	3	0	0	3	2	1	2	0	0	1	2	1	2
MSA 6	0	3	3	2	2	2	0	0	2	0	1	1	0	0	2	0	1	0

*0* normal, *1* mild loss of immunostaining, mild neuronal loss (HE), or mild increase in immunostaining (α-synuclein and p-syn). *2* Moderate loss of immunostaining, moderate neuronal loss (HE), or moderate increase in immunostaining (α-synuclein and p-syn). *3* Marked loss of immunostaining, marked neuronal loss (HE), or marked increase in immunostaining (α-synuclein and p-syn). *NS* no specimen, *Dx* diagnose, *Syn* α-synuclein, *p-syn* phosphorylated α-synuclein, *D-32* DARPP-32, *CALB* calbindin

**Table 3:** Immunostaining intensity in the substantia nigra, pontine nucleus, and the cerebellum

Case no, and Dx	Substantia nigra					Pontine nucleus			Cerebellum			
	HE	Syn	P-syn	CALB^a^	TH	HE	Syn	P-syn	HE	Syn	P-syn	CALB
Control 1	0	0	0	0	0	0	0	0	0	0	0	0
Control 2	0	0	0	0	0	0	0	0	0	0	0	0
Control 3	0	0	0	0	0	0	0	0	0	0	0	0
Control 4	0	0	0	0	0	0	0	0	0	0	0	0
Control 5	0	0	0	0	0	0	0	0	0	0	0	0
ALS 1	0	0	0	0	0	0	0	0	0	0	0	0
ALS 2	0	0	0	0	0	0	0	0	0	0	0	0
ALS 3	0	0	0	0	0	0	0	0	0	0	0	0
ALS 4	0	0	0	0	0	0	0	0	0	0	0	0
ALS 5	0	0	0	0	0	0	0	0	0	0	0	0
ALS 6	0	0	0	0	0	0	0	0	0	0	0	0
MSA 1	3	1	3	3	3	3	1	2	3	2	1	3
MSA 2	2	1	3	3	2	1	3	3	2	3	3	2
MSA 3	3	2	3	3	3	3	3	3	3	2	2	3
MSA 4	NS	NS	NS	NS	NS	NS	NS	NS	3	3	2	3
MSA 5	3	2	3	3	2	3	2	2	3	1	1	3
MSA 6	2	2	3	1	2	2	3	3	2	3	3	2

^a^Dorsal tier of the substantia nigra along the lower edge of the red nucleus. *0* normal, *1* mild loss of immunostaining, mild neuronal loss (HE), or mild increase in immunostaining (α-synuclein and p-syn). *2* Moderate loss of immunostaining, moderate neuronal loss (HE), or moderate increase in immunostaining (α-synuclein and p-syn). *3* Marked loss of immunostaining, marked neuronal loss (HE), or marked increase in immunostaining (α-synuclein and p-syn). *NS* no specimen, *Dx* diagnose, *Syn* α-synuclein, *P-syn* phosphorylated α-synuclein, *D-32* DARPP-32, *CALB* calbindin

DARPP-32 immunostaining was positive in the striatal neurons, but various degrees of immunostaining loss were noted in MSA (Fig. 2). Loss of DARPP-32 immunostaining represents loss of medium-sized spiny neurons. DARPP-32 immunostaining was decreased either markedly or moderately in all cases examined except for MSA 6, where no loss of DARPP-32 was noted in the anterior putamen and caudate head, whereas the posterior putamen showed moderate loss despite no evidence of neuronal loss in HE sections.

Calbindin-D 28k immunostaining was positive in the striatal neurons, neurons in the dorsal tier of the substantia nigra along the lower border of the red nucleus, and cerebellar Purkinje cells in MSA. Results for the other structures studied, including most of the substantia nigra, were negative for calbindin-D 28k immunostaining. Calbindin-D 28k immunostaining was either markedly or moderately decreased in the striatal areas (Fig. 2), except for the anterior putamen and caudate head of MSA 5 and MSA 6, where no loss of neurons was detected by HE (Table 2). In the posterior putamen, marked or moderate loss of immunostaining was noted in calbindin-D 28k, despite only mild or no loss of neurons in HE. In the substantia nigra, marked loss of immunostaining for calbindin-D 28k was noted in the dorsal tier, except for MSA 6 (Table 3). In other areas of the substantia nigra, calbindin-D 28k was not expressed. In the cerebellar cortex, the number of Purkinje cells was markedly reduced in HE and either marked or moderate loss of immunostaining for calbindin-D 28k was observed (Table 3). None of the neurons in the striatum, substantia nigra, pontine nucleus, and cerebellar cortex expressed the 150-kDa spectrin fragment as observed in the controls.

TH immunostaining was either markedly or moderately reduced in all striatal areas studied, except for MSA 6, where there was mild loss in the anterior putamen and no loss in the caudate head; moderate loss of TH immunostaining was noted in the posterior putamen and substantia nigra (Table 2).

### Order of changes in the striatum

Accumulation of p-syn seemed to be the first event in neurodegeneration among the substances studied (MSA 6, Table 2). Although the anterior putamen and caudate head of MSA 6 did not show neuronal loss by HE, moderate increase in p-syn was noted. Furthermore, in MSA 5, although there was no neuronal loss in the anterior putamen and caudate head, losses of immunostaining for DARPP-32 and calbindin-D 28k were moderate and mild, respectively. In MSA 1 and MSA 2, losses of immunostaining for DARPP-32 and calbindin-D 28k were marked and moderate, respectively, although the loss of neurons was only mild in the caudate head. The same trend was also noted in the anterior putamen and caudate head of MSA 1 and MSA 4. Therefore, after accumulation of p-syn in oligodendrocytes, loss of immunostaining for DARPP-32 and calbindin-D 28k followed and finally loss of neurons occurred.

### Postmortem delay and immunostaining

The maximum postmortem delay in the disease control patients was 14 h in ALS 5. Immunostainings for DARPP-32, calbindin-D 28k, and TH were all normal in the putamen. Calbindin-D 28k immunostaining was also normal in the cerebellum. Although two MSA patients had postmortem delays of 15 and 16 h, we assumed that a valid estimation was possible. Girault et al. (1989) analyzed phosphoproteins in the striatum of PD and progressive supranuclear palsy (PSP) is brains with postmortem delays of 24 h or less.

## Discussion

Neuronal loss was most prominent in the posterior putamen of the striatal area in all cases studied, except for MSA 6. All the patients showed moderate to marked neurodegeneration in the cerebellum and pontine nucleus. Alpha-synuclein and/or p-syn were observed in the oligodendrocytes in all the cases; thus, they fulfilled the criteria for MSA. In MSA 4, no pontine specimen remained, but the cerebellum showed marked degeneration.

Glial cytoplasmic inclusions are mainly but not exclusively observed in oligodendrocytes and at times in astrocytes and neurons (Nakazato et al. 1990; Papp et al. 1989). The major component of the inclusions was previously found to be α-synuclein (Wakabayashi et al. 1998). Distribution of this inclusion is widespread in the central nervous system, including the cerebral white matter, basal ganglia, thalamus, brain stem, cerebellum, and spinal cord (Inoue et al. 1997). In the cerebellum, the density of glial inclusions was reported to be more prominent when neuronal loss was mild and almost none when neuronal loss was extensive (Inoue et al. 1997; Masui et al. 2012). In our cases, α-synuclein and/or p-syn were present in all brain areas examined, even in the areas where no neuronal loss was noted by HE staining. Therefore, glial inclusions were thought to be an early-stage phenomenon of the disease. Alpha-synuclein is a neuron-specific protein; therefore, glial α-synuclein must have come from neurons. How neuron-specific protein accumulates in oligodendrocytes is unknown.

DARPP-32 is highly expressed in medium-sized spiny neurons in the striatum containing D-1 receptors and is a modulator of the cAMP-signaling pathway (Walaas and Greengard 1984). All the medium-sized spiny neurons in the striatum were shown to have high concentrations of DARPP-32, and both direct D-1 receptor-expressing neurons and indirect D-2 receptor-expressing neurons expressed DARPP-32 equally (Bertran-Gonzalez et al. 2008). DARPP-32 has been used as a marker protein for medium-sized spiny neurons in the striatum (Ouimet et al. 1984). Immunostaining was more decreased for DARPP-32 than for the morphological changes of striatal neurons in MSA, which suggested that loss of DARPP-32 preceded the loss of neurons in the striatum. Loss of DARPP-32 appears to be not because of the loss of dopamine in the substantia nigra and striatum, because the level of DARPP-32 in PD and PSP has been reported as not being decreased in postmortem striatum (Girault et al. 1989). Furthermore, DARPP-32 levels were unchanged in animals with nigrostriatal dopaminergic lesions (Raisman-Vozari et al. 1990). In MSA, DARPP-32 immunostaining results were reported to be positive in neurons, inclusion-positive oligodendrocytes, and astrocytes. In our study, glial cells were not stained for DARPP-32 (Honjo et al. 2008). DARPP-32-knockout mice do not show a clear clinical phenotype (Fienberg and Greengard 2000); however, altered dopamine modulation of membrane excitability in striatal spiny neurons has been reported (Onn et al. 2003).

Calbindin-D 28k is a vitamin D-dependent calcium-binding protein expressed in many organs, including the brain (Séquier et al. 1988). Calbindin-D 28k immunostaining has been demonstrated in many neurons, particularly in cerebellar Purkinje cells but not in motor neurons (Garcia-Segura et al. 1984). In the basal ganglia, it is expressed in the cell bodies, dendrites, and spines of medium spiny neurons (DiFiglia et al. 1989). In humans, an uneven distribution of calbindin-D 28k within the striatum has been reported; immunostaining was most prominent in the caudate head, moderately weak in the anterior striatum, and much weaker in the posterior striatum (Karachi et al. 2002). Thus, areas showing less immunostaining appear to be more characteristic of MSA.

In MSA, extensive depletion of calbindin-positive medium-sized spiny neurons in the putamen was recently reported (Sato et al. 2007). Loss of calbindin-D 28k has been reported in the Purkinje cells of spinocerebellar degeneration; the authors suggested that loss of calbindin preceded neuronal loss because loss of calbindin-D 28k was noted even in the remaining Purkinje cells (Ishikawa et al. 1995). Wüllner et al. (2000) reported a marked decrease in calbindin-D 28k in the Purkinje cells of MSA, which suggested that a diminished calcium-binding capacity might lead to a change in regulation of proteins of the bcl-2 family. In PD, calbindin immunostaining was reportedly diminished in the ventrolateral tier of the substantia nigra (Damier et al. 1999), where expression of calbindin was diminished. However, most nigral neurons did not express calbindin-D 28k (McRitchie and Halliday 1995), and it seems unlikely that calbindin has a significant role in its pathogenesis. Normal calbindin-D 28k staining in the striatum in PD has been reported (Ito et al. 1992). Loss of calbindin-D 28k has also been reported in Huntington’s disease (Seto-Ohshima et al. 1988) and Alzheimer’s disease (McLachlan et al. 1987; Ichimiya et al. 1988).

In our study, calbindin-D 28k was decreased in the putamen, the dorsal tier of the substantia nigra along the lower border of the red nucleus, and in the cerebellar cortex in MSA. Immunostaining was decreased before the neurodegeneration observed by HE staining in the striatum, substantia nigra, and in cerebellum, which suggested that loss of calbindin-D 28k preceded the loss of neurons. Calbindin-D 28k-knockout mice did not show a clear clinical phenotype; however, alterations in Purkinje cell firing (Servais et al. 2005) and locomotor activity were reported recently (Farre-Castany et al. 2007).

One of the important functions of calbindin is buffering of calcium entry on stimulation of glutamate receptors coupled with calcium channels. Striatal medium spiny neurons receive very strong glutamatergic innervation from the cerebral cortex. The intracytoplasmic concentrations (μM order) of calcium are approximately one-hundredth that of extracellular calcium (mM order). High concentration of intracytoplasmic calcium may activate calcium-dependent protease (calpain) and lead to neurodegeneration. We wanted to evaluate the extent of activation of calpain by immunostaining for the 150-kDa spectrin fragment; however, no immunostaining was detected in neuronal cells, in both MSA and the controls.

More prominent loss of immunostaining for calbindin-D 28k than for degenerative changes is indicative of diminished buffering action to calcium entry. In addition, phosphorylated DARPP-32 is a strong inhibitor of protein phosphatase 1 that regulates the phosphorylated states of many downstream proteins including calcium channel proteins (Greengard et al. 1999); more prominent loss of immunostaining for DARPP-32 than for degenerative changes is indicative of derangement of the functions of these proteins. Further studies appear to be necessary to elucidate the pathogenesis of MSA.

## Abbreviations

DARPP-32: cAMP-regulated phosphoprotein of 32 kDa
MSA: Multiple system atrophy
ALS: Amyotrophic lateral sclerosis
HE: Hematoxylin and eosin
p-syn: Phosphorylated α-synuclein
PBS: Phosphate-buffered saline
PD: Parkinson’s disease
PSP: Progressive supranuclear palsy
TH: Tyrosine hydroxylase
